# Predicting the Potential Distribution of the Szechwan Rat Snake (*Euprepiophis perlacea*) and Its Response to Climate Change in the Yingjing Area of the Giant Panda National Park

**DOI:** 10.3390/ani13243828

**Published:** 2023-12-12

**Authors:** Xinqiang Song, Ying Jiang, Li Zhao, Long Jin, Chengzhi Yan, Wenbo Liao

**Affiliations:** 1 Key Laboratory of Southwest China Wildlife Resources Conservation (Ministry of Education), China West Normal University, Nanchong 637009, China; 2 Daxiangling Provincial Nature Reserve, Ya’an 625200, China; 3 School of Ecology and Nature Conservation, Beijing Forestry University, Beijing 100083, China; 4 Key Laboratory of Artificial Propagation and Utilization in Anurans of Nanchong City, China West Normal University, Nanchong 637009, China; 5 College of Panda, China West Normal University, Nanchong 637009, China

**Keywords:** climate change, *E. perlacea*, environmental variables, distribution, conservation

## Abstract

**Simple Summary:**

This study employs maxent models to investigate the impact of climate change on the potential distribution of the Szechwan rat snake (*Euprepiophis perlacea*) in the Yingjing Area of the Giant Panda National Park. The analysis reveals the significant effects of distance from the streams and slope of the geographic distribution of *E. perlacea*. Furthermore, this study indicates a non-significant reduction in the potential distribution area for the species by the 2050s, which is attributed to reduced anthropogenic activities and habitat fragmentation within the Yingjing Area of the Giant Panda National Park. These findings validate the development of conservation guidelines for *E. perlacea*. More research is needed to identify suitable protected areas and wildlife corridors for *E. perlacea* across its distribution regions, thus enhancing its conservation prospects.

**Abstract:**

Climate change is a significant driver of changes in the distribution patterns of species and poses a threat to biodiversity, potentially resulting in species extinctions. Investigating the potential distribution of rare and endangered species is crucial for understanding their responses to climate change and for the conservation of biodiversity and ecosystem management. The Szechwan rat snake (*Euprepiophis perlacea*) is an endemic and endangered species co-distributed with giant pandas, and studying its potential distribution contributes to a better understanding of the distribution pattern of endangered species. In this study, we confirmed seven presence points of this species in the Yingjing Area of the Giant Panda National Park, and selected eleven key factors to predict the potential distribution of *E. perlacea* under current and future scenarios using MaxEnt models. Our study consistently achieved AUC values exceeding 0.79, meeting the precision requirements of the models. The results indicated that the high potential distribution area of *E. perlacea* is mainly located near Yunwu mountain and the giant panda rewilding and reintroduction base, accounting for approximately 12% of the protected area. Moreover, we identified the primary environmental factors influencing the distribution of *E. perlacea* as the distance from streams and the slope degree, with their contribution rates exceeding 41% and 31%, respectively. In comparison to the current scenario, the potential habitat range for *E. perlacea* did not show an overall reduction in the context of future climate scenarios. To ensure the long-term preservation of *E. perlacea*, it is advisable to validate its actual distribution based on the models’ results. Particular attention should be given to safeguarding its core distribution areas and raising awareness among residents within the potential distribution range about the conservation of *E. perlacea*.

## 1. Introduction

The Earth is currently experiencing the sixth mass extinction event in biodiversity, with over 500 terrestrial animal species teetering on the brink of extinction [1,2]. Amid the myriad of factors contributing to the decline in species populations and extinctions, climate change resulting from human activities has already emerged as a primary and enduring threat to biodiversity [3,4,5,6]. A substantial body of research evidence underscores the profound effects of climate change on various facets of species, encompassing their distribution, phenology, morphology, physiology, and population dynamics [7,8,9,10,11,12]. As an illustration, the annual phenology traits of a Mediterranean snake species (*Vipera aspis*) are undergoing changes due to climate shifts [13]. Species exhibit adaptability to climate change through adaptive evolution and alterations in their distribution ranges. A wealth of evidence suggests that numerous species are undergoing migration toward polar regions and higher altitudes [14,15,16]. Considering the limited dispersal ability of ectothermic organisms and their pronounced temperature dependence, certain studies anticipate a substantial reduction in the potential distribution area of snakes [17,18]. Simultaneously, other research projects anticipate an expansion in the potential range of numerous temperate reptiles in the Northern Hemisphere due to climate change [19,20,21,22]. Notably, some species may be migrating to habitats beyond protected areas, resulting in their disappearance within these designated zones, thereby posing a challenge to the effective conservation of these species.

Climate change is instigating shifts in the geographical distribution, suitable habitats, and phenological patterns of wildlife species [23,24]. These shifts are causing them to move beyond the boundaries and functional zones of nature reserves primarily dedicated to wildlife conservation [25,26]. Consequently, the protective functions of these sanctuaries are at risk of being compromised. Previous studies indicate that the current and future network of protected areas in Morocco may not be sufficient to prevent amphibian and reptile species loss. New protected areas should be considered to protect species identified as highly vulnerable to climate change [27,28]. Meanwhile, a study on snakes in the Brazilian Atlantic Forest hotspot indicates that, by 2080, 73.6% of oviparous species and 67.6% of viviparous species could lose at least half of their original range [17]. The research finds that existing protected areas in the Atlantic Forest Hotspot have limited capacity to safeguard snakes presently, maintaining this precarious protection in the future [17]. Collectively, all the evidence underscores the significant impact of climate change on the biodiversity conservation functions of Nature Reserves, imposing substantial pressures and challenges for preserving biodiversity in these protected areas in the future. Therefore, the analysis and identification of the primary impacts of climate change on species distribution are critically important for understanding and addressing the climate change risks in Nature Reserves.

Rare and endangered species often exhibit limited geographic ranges and specific habitat requirements, rendering them less adaptable to climate change and elevating the risk of habitat loss and heightened extinction vulnerability [29]. Examining the influence of climate change on the distribution of these endangered and rare species holds paramount significance. Species Distribution Models (SDMs) constitute a class of models that utilize species occurrence data and environmental variables to project the potential distribution of species [30,31]. They have arisen as invaluable tools in extensive research on climate change prediction and have become indispensable for investigating how species’ geographic distributions respond to climate change, and for assessing the effectiveness of protected areas under global change [32,33]. Among the array of species distribution models at our disposal, the MaxEnt model, grounded in the maximum entropy principle, distinguishes itself through its precision in outcome prediction and robust spatiotemporal extrapolation capabilities [34]. The MaxEnt model is characterized by several advantages, including its low sensitivity to collinearity among environmental variables, its robustness in situations with limited data points, and its capacity to model intricate relationships among variables [35,36]. Consequently, it has gained widespread and frequent application in recent years within the realms of conservation biology, invasion biology, and other fields that pertain to the impact of global climate change on species distribution and genetic diversity [37]. Of particular significance is the model’s extensive utility in the domain of conservation biology, where it is extensively employed for the assessment of habitat suitability, exploration of species’ ecological niche shifts, and the prognostication of global climate change impacts on the geographical distribution of specific species [38].

The Szechwan rat snake (*Euprepiophis perlacea*), is a species endemic to western China. It holds a critical status as a protected wildlife species in Sichuan Province, being classified as “Critically Endangered (CR)” in the China Red List of Endangered Animals [39]. Furthermore, it is listed as “Endangered (EN)” by the International Union for Conservation of Nature [40]. Since Stejneger named a male specimen collected in Ya’an in 1929 [41], no further discoveries were made for the next half-century, casting doubts on the authenticity of its species. Not until the 1980s did Deng collect a specimen labeled as number 3 in the Wolong Nature Reserve in Wenchuan County and the Hailuogou Forest Glacier Park in Luding County [42]. Zhao compared the differences in hemipenes between the *E. perlacea* and the Mandarin rat snake (*Euprepiophis mandarinus*), discussing the validity of the *E. perlacea* [39]. Chen et al. confirmed the validity of this species through molecular biology research [43]. In 2017, significant breakthroughs were achieved in the artificial breeding of *E. perlacea* [44]. However, this does not signify that *E. perlacea* has completely escaped a precarious situation. Whether *E. perlacea* remains endangered still depends on the stability of its habitat and the stability of the wild population. The sole investigation into the distribution of this species revealed a significant overlap in the distribution range between the *E. perlacea* and the giant pandas (*Ailuropoda melanoleuca*) [45]. The Szechwan rat snake primarily inhabits moist mountainous areas at elevations ranging from 1500 m to 2600 m, with habitats often found under deciduous broad-leaved forests, farmland, grasslands, and shrub thickets [45]. 

As an indicator species for the ecological environment, the presence of the Szechwan rat snake can be considered strong evidence of the excellent ecological conditions in giant panda habitats. However, despite being endangered and rare, the Szechwan rat snake, like most reptiles, has not received proactive protection from humans. Due to its small size and non-venomous nature, the Szechwan rat snake has limited migration and self-defense capabilities [43,44,45]. With the acceleration of habitat development, the Szechwan rat snake is frequently threatened by human activities. During the field investigation conducted in the Yingjing Area of the Giant Panda National Park, it was observed that many local residents, including farmers living around the habitat, adopt a hostile attitude towards non-venomous snakes, including the Szechwan rat snake. There was even a distressing incident where one Szechwan rat snake was found beaten to death. Therefore, the immediate priority is to predict the potential distribution areas of the Szechwan rat snake in the current and future scenarios within this protected area. This is essential for initiating habitat conservation and restoration efforts, aiming to better protect the natural population of the Szechwan rat snake in the wild. Moreover, it extends a range of recommendations and references that contribute to the broader cause of biodiversity conservation within the Yingjing Area of the Giant Panda National Park.

## 2. Materials and Methods

### 2.1. Study Site

**Figure 1:**
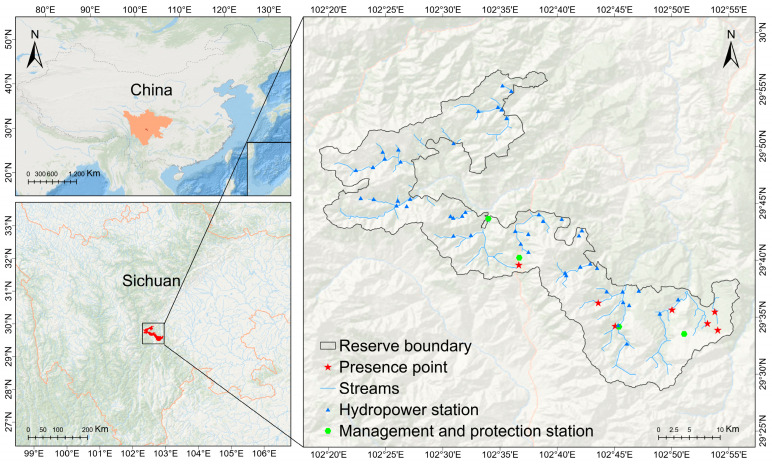
Distribution of presence points of *E. perlacea*.

The study was carried out in Yingjing Area of Giant Panda National Park, China which lies between 102°19′–102°55′ longitude and 29°28′–29°56′ latitude with a total geographical area of 836 km^2^ (Figure 1) [46]. The altitudinal range within the Yingjing Area of the Giant Panda National Park is relatively extensive, with the highest elevation cresting at 3481 m, and the lowest point descending to 1150 m. This locale predominantly features a subtropical monsoon mountainous climate, boasting an annual mean temperature of 16.3 °C, with precipitation levels exceeding 1000 mm and an average frost period of approximately 60 days [47]. The spectrum of vegetation types encompasses evergreen broadleaf forests, soft (and hard) broadleaf forests, mixed coniferous and broadleaf forests, temperate coniferous forests, as well as subalpine shrublands and meadows [46]. This region is crisscrossed by meandering valleys, abounding in abundant water sources, thereby nurturing a lavish tapestry of biodiversity.

The aquatic system within the protected zone is part of the primary tributary of the Qingyi River, known as the Yingjing River basin. Dominant tributaries in this region include the Jing River and the Xiangling River [48]. Within the safeguarded region, the Jing River gives rise to two primary branches: the Heishi River and the Baishi River. The Baishi River spans 34.05 km with a watershed area of 188.8 km^2^, while the Heishi River extends for 31.2 km, covering a watershed area of 219.85 km^2^ [48]. Originating from the northern foothill of Beihou Mountain, situated on the western slope of Xiaoxiangling, is the Xiangling River. Referred to as Huanglian Gou, the upper reaches extend over a length of 25.2 km, encompassing a watershed area of 166.35 km^2^. The natural elevation drop is 2090 m, with a mouth flow measuring 11.5 m^3^/s [48]. 

### 2.2. Input Data

Drawing on the ecological behaviors of the Szechwan rat snake and considering the topography, terrain, and vegetation distribution within the conservation area, this study established a total of 15 effective transects near three conservation stations: Yunwu Mountain, Niba Mountain, and the Giant panda rewilding and reintroduction base (Figure S1). Survey teams, consisting of 2 to 4 individuals per group, conducted meticulous searches along the transects at a speed of 1–2 km/h, covering areas within a 5 m radius on both sides [46]. They recorded discovery locations, habitat types, and species images, utilizing GPS to track their trajectories. Additionally, images of the Szechwan rat snake were provided to conservation area staff or residents for identification, thereby obtaining potential distribution information. Finally, seven occurrence points were successfully obtained.

In the present study, we exclusively utilized seven occurrence points to construct the model. These presence points were meticulously geo-referenced during primary ground surveys using GPS technology. To ensure data quality, the positional accuracy of the occurrence points was verified through Google Earth [49]. Duplicate points were identified and subsequently removed, resulting in the retention of only one point within each 1 × 1 km^2^ grid, a measure taken to mitigate potential sampling bias [49]. Such bias could disproportionately favor the climatic conditions of locations with concentrated sampling efforts [49]. Given the limited number of presence points, which fell below 20, we applied the 1.5 × Interquartile Range (1.5 IQR) method to identify and address potential outliers in the climate data [49]. This approach was based on the environmental data acquired from the WorldClim website at a spatial resolution of 30 arc seconds.

19 bioclimatic variables, encompassing both current (1970–2000) and three future (2050s) scenarios, were obtained from the WorldClim database (http://www.worldclim.org/, accessed on 17 Augst 2023) at a spatial resolution of 30 arc seconds. For the future scenarios, the bioclimatic data for the 2050s represents the mean values from 2041 to 2060. To estimate future climate change, we utilized predictions from general circulation models (GCMs) based on the Shared Socio-economic Pathways (SSPs) scenarios, introduced as part of the Coupled Model Intercomparison Project Phase 6 (CMIP6) by the IPCC [50]. The GCM we chose was the BCC-CSM2-MR climate system model which was developed by the National Climate Center [51]. Three scenarios, SSP 1-2.6, SSP 2-4.5, and SSP 5-8.5, were chosen in this study.

Additionally, we employed the WGS84 projection and acquired a nationwide 30 m × 30 m digital elevation model (DEM) dataset from the National Aeronautics and Space Administration [52]. With this dataset, we conducted calculations to determine slope, aspect, and the Euclidean distance from each grid cell to the nearest stream (distance from stream) based on the DEM data [53]. The soil water regime data were sourced from the Harmonized World Soil Database version 1.2 [54]. The vegetation data correspond to the Normalized Difference Vegetation Index (NDVI), which was produced using MODIS/Terra (MOD13C1 and MOD13C2). To ensure synchronization with the survey’s timeframe, we opted for the July 2022 dataset. All environmental data underwent a thorough cross-check for resolution accuracy and were adjusted to a 30 arc second pixel resolution.

### 2.3. Ecological Niche Modeling (ENM)

All modeling was conducted using MAXENT Version 3.4.3, given the reliance on presence points exclusively and the limited sample size [49,55]. MAXENT is specifically designed to effectively accommodate small samples. The model was constructed using the Jackknife method [56]. To validate the model’s robustness, we executed seven replicated model runs, employing a threshold rule of the 10th percentile for training presence and a cross-validation technique to partition the samples into replicate folds, with the remaining data serving as the test dataset, while keeping all other parameters at their default values [49]. For instance, in the default state, 10,000 points were randomly chosen as background points. Furthermore, recognizing the potential for model instability when dealing with a multitude of factors, we conducted an initial simulation using 25 environmental variables. The aim was to identify the factors with a relatively significant contribution. Subsequently, a secondary simulation was conducted. Three methods, namely True Skill Statistic (TSS), Cohen’s Kappa (Kappa), and Area Under the Curve of Receiver Operator Characteristic (ROC) curves (AUC), were employed to assess the predictive accuracy of the model [53]. Utilizing ArcGIS 10.8’s transformation tool, we converted the average results of the seven model predictions from ASCII-encoded files to a raster format [49]. These results were subsequently reclassified using manual grading techniques. Considering the maximum training sensitivity plus specificity threshold (range from 0.46 to 0.54) and the natural intermittent classification method (Jenks), we opted to categorize the distribution potential of the model into five classes: very low (0–0.2), low (0.2–0.4), medium (0.4–0.6), high (0.6–0.8), and very high (0.8–1) [49,52].

## 3. Results

The mean AUC values for the model’s predictions of *E. perlacea* under different climatic scenarios were consistently greater than 0.79 (Table 1). The model exhibited a strong performance, meeting the accuracy requirements, and demonstrated relatively good stability over ten repeated runs. However, the mean TSS and Kappa values were comparatively low (Table S1). We attributed this to the relatively small size of presence points and the use of background points instead of true absence points [57]. Additionally, it has been demonstrated in studies that in cases with a limited number of presence points, the AUC value is the optimal metric to evaluate the effectiveness of the model [58]. Consequently, the MaxEnt model utilized in this study maintains high accuracy and reliability in predicting the distribution range of *E. perlacea* under different climate scenarios.

The results revealed that the distribution of *E. perlacea* was influenced by 11 environmental factors, each with varying degrees of contribution (Table 2). Notably, the Euclidean distance to streams and the slope gradient exhibited relatively high contributions, each exceeding 41% and 31%, respectively. The mean diurnal range (Bio2) and isothermality (Bio3) follow in terms of their relative contributions; the remaining variables make minor contributions. It is evident that the 11 bioclimatic variables displayed differences in their contributions to the modeling of *E. perlacea*. Additionally, *E. perlacea* was predominantly found in low-lying mountainous regions, often associated with the presence of streams.

**Figure 2:**
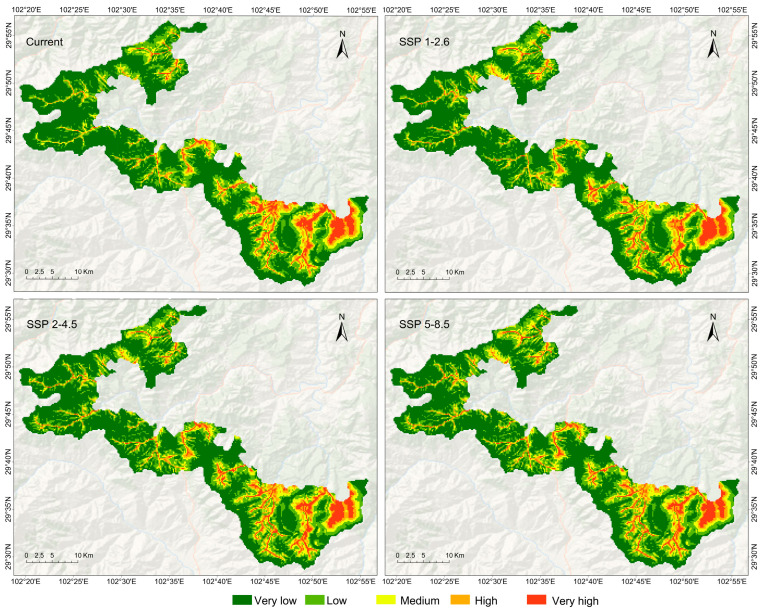
The potential distribution of *E. perlacea* in Yingjing Area of the Giant Panda National Park under different scenarios.

The results obtained from the MaxEnt model revealed that the potential distribution area of *E. perlacea* was predominantly situated in the eastern region of the Yingjing Area of the Giant Panda National Park, particularly in areas with streams near Yunwu mountain and the Giant panda rewilding and reintroduction base (Figure 2). In comparison to the present, there was no conspicuous trend of significant reduction in the potential distribution area of *E. perlacea* for the 2050s (Table 3).

## 4. Discussion

Our study on the potential distribution of *E. perlacea* in the Yingjing Area of the Giant Panda National Park provides novel insights into the significant effect of the distance from streams, the slope degree and the mean diurnal range on distribution. We found that individuals lived in low-lying mountainous regions associated with the rivers. Moreover, this species preferred the potential distribution area situated in the eastern region of the National Park, and we found no trend in significant reductions in distribution area for the 2050s, suggesting no significant effect of climate change on the potential distribution of this species in the Yingjing Area of the Giant Panda National Park.

The MaxEnt model was selected to develop predictive models despite the constraints of a limited sample size within a smaller area of 836 km^2^ for forecasting the potential distribution of *E. perlacea*. The MaxEnt model is notably valuable for assessing the current and potential distributions of rare, threatened, and poorly known species, particularly among various snake species. Consequently, it has gained widespread acceptance as an essential tool in systematic conservation planning and management [59]. Applying the MaxEnt model has yielded notable outcomes, such as a comprehensive understanding of 39 species of New World coral snakes spanning North, Central, and South America [60]. Additionally, the model has facilitated predictions regarding the potential distribution for many snake species, accompanied by an evaluation of the conservation status of existing protected areas for the species based on potential distribution assessments [61,62,63,64,65]. Within the context of our study, it is evident that the MaxEnt models for *E. perlacea* consistently achieve AUC values exceeding 0.79, underscoring how our study has, for the first time, established reliable models for predicting the potential distribution of *E. perlacea*. It is essential to acknowledge that our study does not account for factors such as human interference, the influence of natural predators, or competition for food resources, which can exert varying degrees of influence on the potential distribution of *E. perlacea*. Consequently, the model’s results predominantly portray a distribution that aligns with the fundamental ecological niche requirements of *E. perlacea* and may, to some extent, exceed the actual species distribution [66]. Moreover, the MaxEnt model solely predicts static ecological niches, overlooking the gradual adaptation of species to their environment throughout the evolutionary process [67]. The potential distribution of a species hinges on its intrinsic adaptability and dispersal ability, signifying that species distribution is genuinely a dynamic ecological niche [67,68]. Therefore, to achieve a thorough forecast of suitable habitats for *E. perlacea*, it is imperative to incorporate pertinent biological factors. Future research should address the challenges related to quantifying specific indicators and collecting additional data, incorporating various factors influencing species distribution to enhance the precision of species distribution modeling.

The model yielded valuable insights on the topographic factors which are influencing the potential distribution of *E. perlacea.* Notably, it identified the distance from streams and the slope degree as the most important predictors, aligning with previous findings that individuals preferred living near streams and low degrees of sloping [45,69,70]. In a study predicting the potential distribution of six snake species, it was also found that the primary explanatory variable for species distribution was proximity to streams and a low slope degree [69]. Meanwhile, in a separate study focusing on the Pygmy Rattlesnake (*Sistrurus miliarius*), a locally rare snake species, it was observed that *S. miliarius* primarily inhabited riverine or riparian habitats associated with the lower Tennessee river valley [70]. Interestingly, our investigation found that areas close to rivers and lowlands were also suitable for *E. perlacea*, and were often connected to riverine or riparian regions. These findings underscore the potential significance of riparian corridors as suitable habitats for snakes. However, it is essential to acknowledge that riparian ecosystems are susceptible to anthropogenic disturbances, including hydropower stations, pollution and development [71], which may adversely affect *E. perlacea* populations if suitable habitats are indeed positively associated with riparian ecosystems. To safeguard the critical habitat of *E. perlacea* throughout the Yingjing Area of the Giant Panda National Park, it is crucial to consider the preservation of current river water levels and the measures required to mitigate the loss of riparian habitats due to hydrological alterations.

Reptiles are poikilothermic organisms that experience daily fluctuations in body temperature, which is affected by environmental factors. Temperature is regarded as the most important environmental factor which affects the potential geographical distribution and survival of species [72,73]. For instance, the minimum temperature of the coldest month influences the potential geographical distribution of insects [74,75]. However, we did not find a significant effect of the minimum temperature of the coldest month on the potential geographical distribution of *E. perlacea*. Precipitation is also regarded as an important effect on potential geographical distribution because the change in the soil moisture in fields is associated with an increase in potato damage [76]. For instance, a previous study using the MaxEnt model to predict the potential geographical distribution has shown that precipitation seasonality affects the potential geographical distribution of *Phenacoccus solenopsis* in India [77]. In this study, precipitation did not affect the potential geographical distribution of *E. perlacea*. Previous studies have shown that climate change drives changes in the variation of temperature and food resources, and thus the population dynamics and potential geographical distribution [6,78,79,80]. Indeed, there is evidence that variation in temperature affects the potential geographical distribution of reptiles because extreme temperature leads to declining population density [81]. Meanwhile, significant variation in temperature in the Yingjing Area of the Giant Panda National Park will lead to a decrease in the population, thus declining the number of regions of potential distribution. Our results provided evidence that the mean diurnal range was one significant variable affecting the potential geographical distribution of *E. perlacea*.

There is increasing evidence that an increase in the emissions of greenhouse gases causes an increase in global average temperature and global climate warming [82]. As a result, global climate change leads to variation in the distribution of many species. For instance, the distribution range of *Xanthium italicum* will decrease in the future due to climate change [83]. Della et al. [84] have revealed that global climate change was positively associated with the distribution of four species. On the contrary, global climate change is negatively correlated with the distribution of insects, birds and mammals [49,85,86]. The fates of potential geographical distribution of species are decided by their capacity to cope with an increase in temperature and variation in precipitation. Indeed, variation in temperature and precipitation is related to the total number of highly suitable habitat areas, thus affecting potential geographical distributions. In this study, we found there was non-significant reduction in the potential distribution area for the 2050s, suggesting that temperature and precipitation seasonality cannot change the potential geographical distribution of *E. perlacea* in future. 

The models predict that the potential habitats (medium, high, and very high) for *E. perlacea* do not exceed 26% (217 km^2^) of the Yingjing Area of the Giant Panda National Park. Hence, the *E. perlacea*’s habitats in the Giant Panda National Park were far fewer than the average area for the wild animals in the area. While protected areas can effectively confirm the threats from human activities for endangered species, many species facing climate change scenarios possibly shift their distribution ranges outside the protected areas [87,88]. Meanwhile, habitat fragmentation is likely to amplify the constraints on species distribution range shifts by hampering young dispersal [89,90,91]. Those patterns underscore the need to establish valuable protected areas for endangered species under climate change [92]. For *E. perlacea*, this means that anthropogenic activities and habitat fragmentation under climate change are likely to affect their habitats and potential distributions. However, all distribution locations of *E. perlacea* in the Yingjing Area of the Giant Panda National Park have less anthropogenic activity and habitat fragmentation. Hence, we suggest that the conservation of *E. perlacea* in the other distribution regions should rely on building small protected areas and wildlife corridors. 

## 5. Conclusions

We explored the consequences of climate change on the potential distribution of *E. perlacea*, an endangered species that is challenging to observe in the field. However, we cannot delimit the boundaries of protected areas due to insufficient data on a single species. Although we found that there are significant effects of distance from streams, slope degree and mean diurnal range on geographical distribution, more similar studies on building small protected areas and wildlife corridors in the other distribution regions of *E. perlacea* are needed to confirm the most suitable protected areas. At the same time, we found a non-significant reduction in the potential distribution area for the 2050s because less anthropogenic activity and habitat fragmentation in the Yingjing Area of the Giant Panda National Park benefits species conservation. Furthermore, the non-significant variation in distribution areas in *E. perlacea* experiencing climate change is likely to be associated with the refugia regions established through climate change. Nevertheless, there is a probability that other climatic and non-climatic factors, such as biotic interactions, dispersal abilities, and evolutionary adaptations, not considered in our models, influence the spatial distribution of this species. Our future plans thus involve integrating multiple models and considering various factors influencing species distribution. This aims to conduct a more in-depth analysis of *E. perlacea* habitat suitability, offering guidance for *E. perlacea* habitat protection and core reservation planning. Lastly, from a conservation standpoint, there is cause for concern as residents within the potential distribution range currently demonstrate inadequate awareness and protective measures for *E. perlacea*. Consequently, we strongly encourage personnel in conservation areas to not only enhance the protection of the native habitat of *E. perlacea* but also to engage in educational outreach to local residents.

## Figures and Tables

**Table 1:** AUC value of MaxEnt model under different scenarios.

Different Scenarios	AUC Value *
Current	0.83 ± 0.16
2050s SSP 1-2.6	0.81 ± 0.21
2050s SSP 2-4.5	0.80 ± 0.21
2050s SSP 5-8.5	0.79 ± 0.21

* The evaluation criteria for AUC are: Excellent, 0.9–1; Good, 0.8–0.9; Fair, 0.7–0.8; Poor, 0.6–0.7; Failed, 0.5–0.6.

**Table 2:** Relative contribution of environmental variables under different scenarios.

Environmental Variable	Current	2050s		
		SSP 1-2.6	SSP 2-4.5	SSP 5-8.5
Distance from stream	41.90	48.80	48.50	49.50
Slope degree	32.00	37.50	38.80	40.40
NDVI	0.40		0.30	0.30
Soil water regime	0.10	0.40	0.40	0.50
Bio2 (mean diurnal range)	4.30	8.30	12.10	7.70
Bio3 (isothermality)	19.90	0.60		1.10
Bio7 (temperature annual range)				0.10
Bio14 (precipitation of driest month)	1.40			0.10
Bio15 (precipitation seasonality)	0.10	3.90		0.20
Bio17 (precipitation of driest quarter)		0.40		0.30
Bio19 (precipitation of coldest quarter)		0.20		

**Table 3:** Percentage of potential areas for *E. perlacea* in different scenarios in Yingjing Area of Giant Panda National Park.

Distribution Potential	Current	2050s		
		SSP 1-2.6	SSP 2-4.5	SSP 5-8.5
very low	53.59	51.88	50.45	49.56
low	23.30	24.06	24.95	25.40
medium	10.64	12.02	11.95	12.27
high	6.90	6.91	7.14	7.21
very high	5.58	5.14	5.50	5.56

## Data Availability

The data presented in this study are available on request from the corresponding author. The data are not publicly available due to privacy or ethical restrictions.

## Supplementary Materials

The following supporting information can be downloaded at: https://www.mdpi.com/article/10.3390/ani13243828/s1, Figure S1: Map depicting the distribution of management and protection stations and transects in Yingjing Area of Giant Panda National Park; Table S1: The basic information for Maxent result.
