# Supplementary material for: Predicting the Potential Distribution of the Szechwan Rat Snake (Euprepiophis perlacea) and Its Response to Climate Change in the Yingjing Area of the Giant Panda National Park
Source: Animals (Basel). 2023 Dec 12;13(24):3828. doi: 10.3390/ani13243828 (PMC10740900; doi:10.3390/ani13243828)
Supplement: Supplementary file 1 [file animals-13-03828-s001.zip › animals-2720285-supplementary.pdf]

Supporting Information for

Predicting the potential distribution of Szechwan rat snake (*Euprepiophis perlacea*) and its response to climate change in Yingjing area of Giant Panda National Park

This file includes:

Figure S1

Table S1

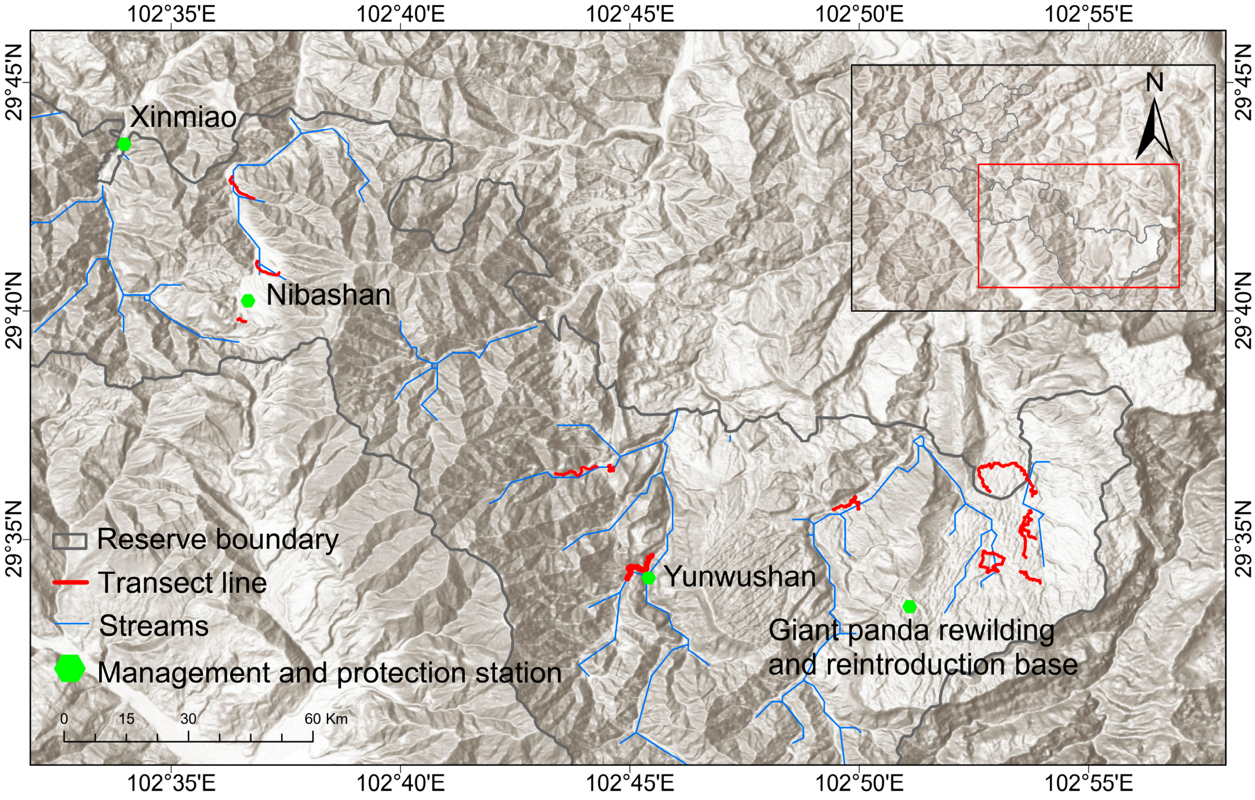

Figure S1. Map depicting the distribution of management and protection stations and transects in Yingjing area of Giant Panda National Park.

Table S1. The basic information for Maxent result.

Replicate	Current			SSP-1.26			SSP-2.45			SSP-5.85		
model	AUC	TSS	Kappa	AUC	TSS	Kappa	AUC	TSS	Kappa	AUC	TSS	Kappa
Model 1	0.7448	-0.2505	0.0006	0.7452	-0.1768	0.0006	0.7117	-0.1981	0.0005	0.6965	-0.1822	0.0005
Model 2	0.9712	0.7276	0.0067	0.9712	0.7771	0.0067	0.9694	0.7602	0.0063	0.9602	0.786	0.0048
Model 3	0.9479	0.7584	0.0036	0.9133	0.814	0.0021	0.9261	0.7833	0.0025	0.9091	0.8179	0.0020
Model 4	0.5355	-0.1325	0.0002	0.3508	-0.113	0.0001	0.3281	-0.1385	0.0001	0.3785	-0.1311	0.0001
Model 5	0.6918	-0.0766	0.0004	0.7141	-0.2503	0.0005	0.6753	-0.0931	0.0004	0.6538	-0.0909	0.0004
Model 6	0.9845	0.7062	0.0125	0.9727	0.8064	0.0071	0.9783	0.747	0.0089	0.9721	0.7972	0.0069
Model 7	0.9634	0.7096	0.0052	0.9829	0.7737	0.0114	0.9744	0.748	0.0076	0.9670	0.7657	0.0058

TSS: True Skill Statistic; Kappa: Cohen's Kappa; AUC: Area Under the Curve of Receiver Operator Characteristic Curves. The evaluation criteria for TSS are as follows: Excellent, 1—0.85; Very good, 0.7—0.85; Good, 0.55—0.7; Fair, 0.4—0.55; Poor, <0.4. The evaluation criteria for Kappa are: Excellent, 1—0.85; Very good, 0.7—0.85; Good, 0.55—0.7; Fair, 0.4—0.55; Poor, <0.4. The evaluation criteria for AUC are: Excellent, 0.9—1; Good, 0.8—0.9; Fair, 0.7—0.8; Poor, 0.6—0.7; Failed, 0.5—0.6.
